# Applications of artificial intelligence for adolescent idiopathic scoliosis: mapping the evidence

**DOI:** 10.1007/s43390-024-00940-w

**Published:** 2024-08-17

**Authors:** Samuel N. Goldman, Aaron T. Hui, Sharlene Choi, Emmanuel K. Mbamalu, Parsa Tirabady, Ananth S. Eleswarapu, Jaime A. Gomez, Leila M. Alvandi, Eric D. Fornari

**Affiliations:** 1 Albert Einstein College of Medicine, 1300 Morris Park Avenue, Bronx, NY 10461 USA; 2 Department of Orthopaedics, Montefiore Medical Center, Bronx, NY 10461 USA

**Keywords:** Artificial intelligence, Machine learning, Adolescent idiopathic scoliosis, Spinal deformity, Orthopaedic surgery

## Abstract

**Purpose:**

Adolescent idiopathic scoliosis (AIS) is a common spinal deformity with varying progression, complicating treatment decisions. Artificial intelligence (AI) and machine learning (ML) are increasingly prominent in orthopedic care, aiding in diagnosis, risk-stratification, and treatment guidance. This scoping review outlines AI applications in AIS.

**Methods:**

This study followed PRISMA-ScR guidelines and included articles that reported the development, use, or validation of AI models for treating, diagnosing, or predicting clinical outcomes in AIS.

**Results:**

40 full-text articles were included, with most studies published in the last 5 years (77.5%). Common ML techniques were convolutional neural networks (55%), decision trees and random forests (15%), and artificial neural networks (15%). Most AI applications in AIS were for imaging analysis (25/40; 62.5%), focusing on automatic measurement of Cobb angle, and axial vertebral rotation (13/25; 52%) and curve classification/severity (13/25; 52%). Prediction was the second most common application (15/40; 37.5%), with studies predicting curve progression (9/15; 60%), and Cobb angles (9/15; 60%). Only 15 studies (37.5%) reported clinical implementation guidelines for AI in AIS management. 52.5% of studies reported model accuracy, with an average of 85.4%.

**Conclusion:**

This review highlights the applications of AI in AIS care, notably including automatic radiographic analysis, curve type classification, prediction of curve progression, and AIS diagnosis. However, the current lack of clear clinical implementation guidelines, model transparency, and external validation of studied models limits clinician trust and the generalizability and applicability of AI in AIS management.

**Supplementary Information:**

The online version contains supplementary material available at 10.1007/s43390-024-00940-w.

## Introduction

Adolescent idiopathic scoliosis (AIS) is a spinal deformity affecting adolescents, characterized by a coronal curvature of the spine > 10°, along with rotation of the vertebrae, and usually reduced kyphosis in the thoracic spine [1]. The worldwide prevalence of AIS varies from 0.47 to 5.2%, emphasizing the need for efficient diagnosis and treatment [2]. Conservative management, such as bracing, is typically recommended for skeletally immature patients with curves ranging from 25 to 45 degrees, while surgery is reserved for severe curves exceeding 45 degrees [3, 4]. AIS patients exhibit diverse rates of curve progression, complicating treatment strategies for physicians [5]. Various factors including skeletal maturity, age, curve location (thoracic vs. lumbar), Cobb angle at initial visit, and flexibility influence curve progression rates [5, 6]. However, attempts to predict curve progression based solely on these factors have shown limited accuracy [5]. As such, a major concern for orthopedic surgeons managing AIS patients with minor curvature is identifying which patients are high-risk to progress to severe deformity and require surgical intervention [7–9].

The use of artificial intelligence (AI) and machine learning (ML) in orthopedic care has rapidly expanded, demonstrating potential to improve diagnostic accuracy and inform treatment decisions [8]. Such applications include, automated osteoarthritis (OA) grading, tumor classification in orthopedic oncology, and fracture detection/classification in orthopedic trauma [9]. Thus, there is optimism AI can improve the complicated management of AIS through improved screening protocols and diagnostic accuracy, prognostic curvature predictions, and creation of patient-specific rehabilitation goals [10–12].

**Fig. 1:**
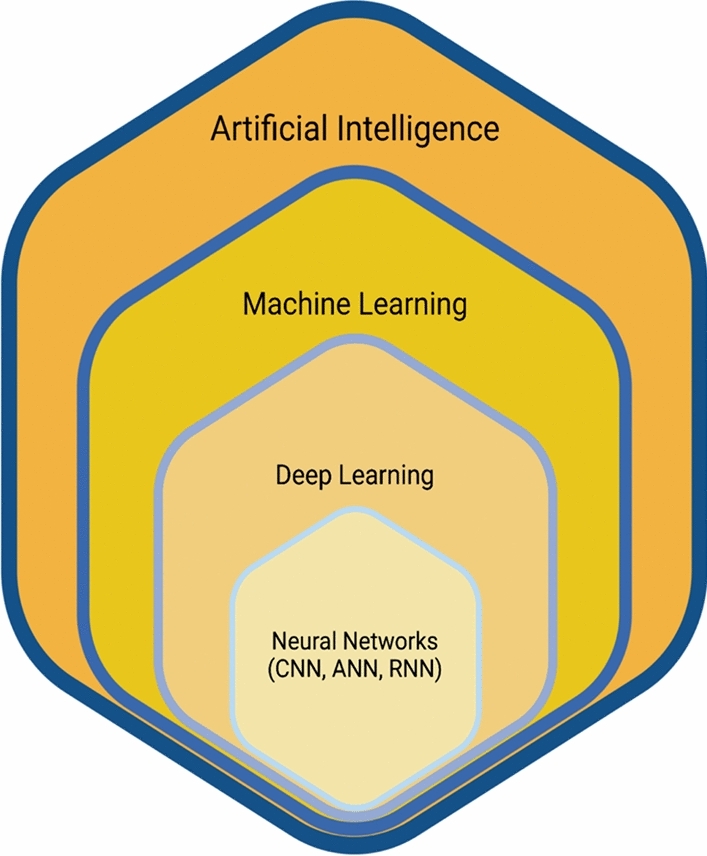
Nested doll analogy for artificial intelligence

Despite the potential benefits of AI for orthopedic care, adoption by surgeons has been hindered by a lack of understanding of the technical processes involved [13]. Although the relevant literature often uses the terms “artificial intelligence,” “machine learning,” and “deep learning” as if they are interchangeable, our team’s prior review proposed a simplified view on AI through a nested doll analogy (Fig. 1) [8]. AI is an all-encompassing term used to describe either machines or algorithms that aim to mimic human cognitive functions, especially for learning and problem-solving. AI uses ML to learn from experience through large data sets to make predictions [14]. Training data sets are used to help the ML algorithm “learn” from patterns in the data. Subsequently, validation data sets are utilized to evaluate the model’s accuracy, following the learning period. The ML algorithm training may be supervised, where the human researchers classify the data ahead of time, or unsupervised, where no data are labeled by humans and the machine recognizes patterns with no goal output values [15]. The overall goal is to both analyze and determine relationships using pattern detection within the training data and ultimately refine its predictions against the testing set through iterative positive or negative reinforcement.

Within ML is deep learning (DL), a subset of ML that employs artificial neural networks (ANNs) and convolutional neural networks (CNNs). ANNs are the simplest type of computational model that mimics the structure and function of neurons in the human brain. ANNs, also called feed-forward networks, consist of interconnected nodes, which each perform a simple mathematical function [16]. The output of one node is the input for the next node, a unidirectional process which repeats until the final nodal layer outputs a prediction. Advantages of ANNs are their adaptability, ability to perform multiple tasks at once, and capacity to output predictions with incomplete data sets. Disadvantages include their limited transparency due to the unexplained behavior of the network. Applications of ANNs include image classification (facial recognition) and speech to text translators on iPhones. CNNs are composed of convolutional, pooling, and fully connected layers. The convolutional layer creates 2D feature maps from input images, the pooling layer reduces the dimensionality of the input data while maintaining critical information, and the fully connected layer integrates the various features from the pooling and convolutional layers through bidirectional maps and generates a final prediction. In other words, the first two layers (convolutional and pooling) aid in image feature extraction, while the third layer (fully connected) classifies the extracted features into a finalized output. Advantages of CNNs are their ability to detect features from images and videos without human supervision, while some challenges include extracting “hidden” details and analyzing images with imperfect orientation [17]. CNNs are typically applied through Computer Vision which is an AI field that allows for data analysis through input of images or videos.

Despite its potential benefits, AI research in AIS is still in its early stages, with most studies emerging in the last five years. Many orthopedic surgeons have a limited understanding of ML techniques, hindering trust and clinical implementation. This scoping review aims to (1) outline the current applications of AI in AIS management, (2) identify commonly used ML techniques, input/output variables, and model performance metrics, (3) evaluate the performance of these models, and (4) identify gaps in the existing AI in AIS literature. Our goal is to highlight AI models that orthopedic surgeons can use to assist in AIS management and suggest directions for future AI-driven AIS research.

## Methodology

This scoping review was performed in accordance with the Joanna Briggs Institute (JBI) methodology for scoping reviews [18]. This review adhered to the Preferred Reporting Items for Systematic Reviews and Meta Analyses extension for Scoping Reviews (PRISMA-ScR) [19]. Our study did not require Institutional Review Board oversight since it did not include human subjects.

### Formulation of research question

We sought to answer the following research questions: (1) How is AI applied to manage AIS? (2) What type of AI and ML models are commonly employed to manage AIS? (3) What were the input and output variables for each AI model? (4) How are the AI models trained and validated? (5) What is the accuracy of the AI models and how is performance assessed? (6) Do studies report on clinical implementation and external validation of these models?

### Literature search strategy and inclusion/exclusion criteria

A literature search of potential studies was conducted across three electronic databases: Web of Science Core Collection (1985–present) via Clarivate Analytics, PubMed via National Library of Medicine, and Embase via Embase.com for any entries from inception until April 2023. Search results were limited to pediatric populations (< 18 years old), human studies, and English language studies. Any article that reported the development, use, and/or validation of an AI model in pediatric orthopedic patients with AIS were included. Letters to the editor, conference abstracts, technique papers, animal and cadaveric studies, non-English studies, review articles, meta-analyses, overviews, and systematic reviews were excluded. A preliminary search guided by our institution’s librarian, was conducted using the MEDLINE and Embase databases. Based on keywords found in titles, abstracts, and texts from this preliminary search, a comprehensive search strategy was developed. A combination of Medical Subject Heading ([MeSH] for PubMed and Emtree for Embase) terms (or equivalent) and free text were searched in the title and abstract fields. Literature identified from these searches were managed using the Covidence software platform (Veritas Health Innovation Ltd, Melbourne, Australia). The complete search strategy and search terms used are demonstrated in a Supplemental file.

### Study selection

The abstract screening and full-text reviews were performed and assessed independently by four investigators (AH, SG, ME, and PT). Each abstract and full text article was assessed by two independent reviewers. There was full agreement on the studies selected for inclusion. Conflicts between reviewers were resolved through a majority voting process.

### Data extraction

Data were extracted from eligible studies into an evidence table to summarize the following: year of publication, the function of the AI, the type of AI model, input and output features, radiographic features or parameters extracted (Cobb angle, apical vertebrae, etc.), mean Cobb angle, integration of skeletal maturity in AI model (Y/N), AI validation technique, model performance accuracy, model performance evaluation metrics, training set sample size, validation set sample size, mention of clinical implementation, country of study, mean age of patients in the study, and study conclusions.

Each full-text article had data extraction performed by two or more independent reviewers (AH, SG, SC, EM, PT) using a customized data extraction tool from Covidence. Following extraction, consensus between reviewers was achieved through group discussion. After consensus, a final data extraction sheet was exported to excel for analysis.

### Data analysis

Given the inherent limitations of a scoping review, a formal, pooled analysis could not be conducted. Instead, descriptive statistics (frequencies and percentages) were utilized.

## Results

### Study characteristics

**Fig. 2:**
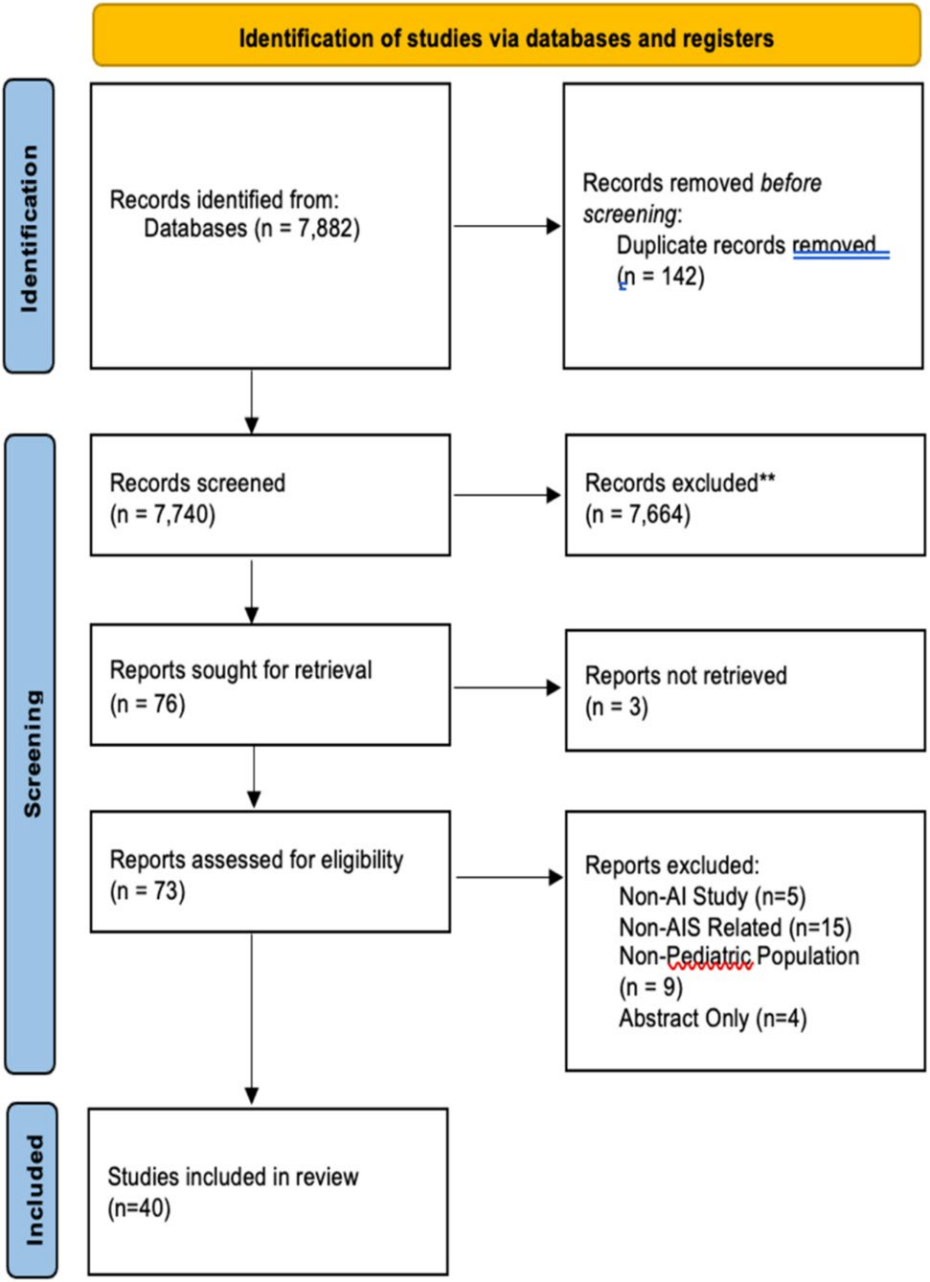
PRISMA diagram

**Fig. 3:**
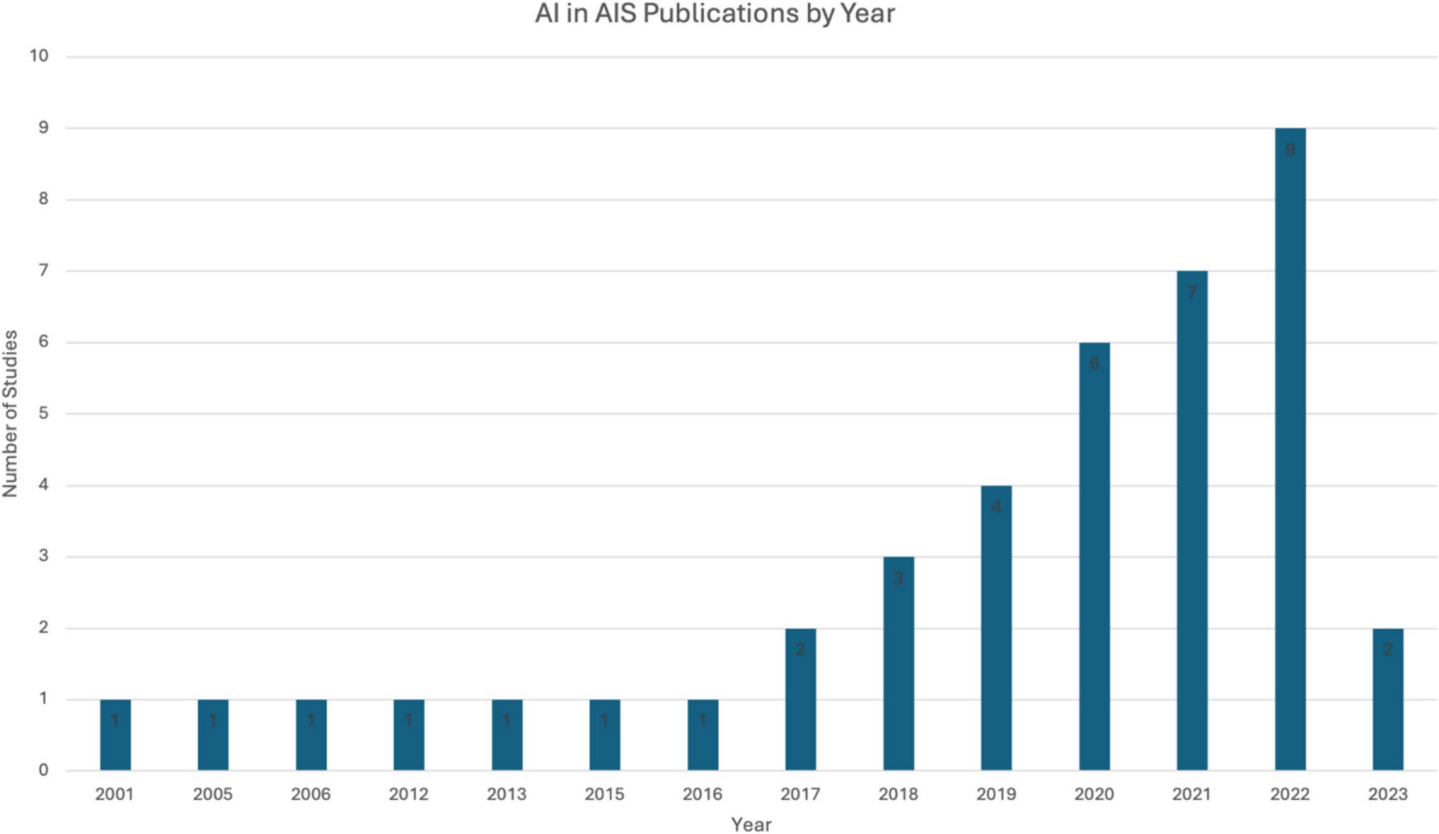
Temporal distribution of published articles on AI in AIS

The initial literature search retrieved 7882 articles. Following the full-text review, 40 studies were included (Fig. 2). A summary of the primary extracted variables and conclusions, categorized by the study’s application of AI, is presented in Table 1 Most articles (31/40, 77.5%) were published in the last 5 years, and 90% of studies were published in the last decade (Fig. 3). All studies were published from economically developed countries such as Canada (18/40, 45%), China (9/40, 22.5%), Japan (4/40, 10%), United States (3/40, 7.5%), Germany (2/40, 5%), United Kingdom (2/40, 5%), Australia (1/40, 2.5%), and France (1/40, 2.5%).

**Table 1:** Extracted variables from included studies categorized by AI application in AIS management

Study ID	AI type	AI application	Input parameters	Radiographic output parameters	AI validation method	Training set sample size	Model performance	Conclusion
Berlin [20] 2023	CNN	Radiographic analysis (automatic measurement)	Imaging (radiographs, CT, MRI, ultrasound)	Cobb angle; apical vertebra; T1-tilt; Clavicle angle; coronal balance	Not specified	100	ICC = 78–98% RMSE = 0.7–8.3 degrees	The authors developed an AI-based algorithm for coronal radiographic parameters that alleviates physicians from time-consuming routine work and error-prone measurements
Huang [42] 2022	CNN	Radiographic analysis (automatic measurement)	Imaging	Cobb angle; apical vertebra; vertebra identified (such as T1-L5)	Cross validation not otherwise specified	200	ICC = 96.7% MAE = 2.9 degrees	The authors created a fully automated framework that identifies the vertebral boundaries and calculates the Cobb angle of PT, MT, and TL/L curves sequentially, increasing efficiency in AIS management
Logithasan [43] 2022	CNN	Radiographic analysis (automatic measurement, spinal segmentation)	Imaging	Cobb angle; axial rotation; apical vertebra; T1-tilt	Cross validation not otherwise specified	55	Accuracy = 81%	The authors used CNNs to automatically measure axial vertebral rotation (81% accuracy) based on the Stokes method on PA radiographs for children with AIS
Zhang [44] 2021	CNN	Radiographic analysis (automatic measurement, spinal segmentation)	Imaging	Cobb angle; vertebra identified (such as T1-L5); Clavicle Angle	Not specified	609	Landmark estimation MSE = 0.004 Cobb angle estimation SMAPE = 25.99	The authors presented a novel method to estimate the spinal landmarks and Cobb angles from grayscale PA X-ray images. Clinicians can determine the Cobb angles of a spinal X-ray image accurately, improving AIS treatment
Magnide [45] 2021	CNN and SVM	Radiographic analysis (automatic measurement)	Imaging	Not Specified	FCV	53	Accuracy = 79% PPV = 80% Sensitivity = 79.3% F1 Score = 80%	The authors proposed a machine learning approach to incorporate many image features for different regions of interest to improve Risser grading for skeletal maturity
Rothstock [46] 2020	ANN	Imaging analysis (automatic measurement, curve severity, decreased radiation exposure)	Imaging, 3D modeling	Cobb angle	Cross validation not otherwise specified	50	Accuracy = 90% Sensitivity = 100% Specificity = 80%	A semi-automatic method for the classification of scoliosis severity and treatment group using 3D marker-less ST scans was proposed by the authors. Classification success rate for scoliosis severity was 90% with a sensitivity of 80% and specificity of 100% for a mild and moderate-severe patient group
Gardner [47] 2021	KNN	Radiographic analysis (automatic measurement, curve classification)	Live camera; 3D modeling	Kyphosis angle	Not specified	691	Accuracy = 93%	The authors’ work shows that there are 5 different types of Lenke 1 curve when assessed using the parameters of scoliosis curve size, kyphosis and the amount of torso asymmetry. Using the k-nearest neighbor algorithm, these clusters can be identified with accuracy in an automated fashion
Thong [48] 2016	ANN	Radiographic analysis (automatic measurement, curve classification/severity)	Imaging, 3D modeling	Cobb angle; Kyphosis angle; Lumbar Lordosis angle; axial rotation; apical vertebra	Not specified	645	N/A	In this 3D analysis of spinal deformities, a novel method simplifying the representation of the geometric 3D reconstruction of a patient’s spine was presented. An automated classification method, called stacked auto-encoders, discovers sub-groups within a large pool of patients with both thoracic and lumbar AIS deformations
Jaremko [49] 2001	ANN	Imaging analysis (automatic measurement, decreased radiation exposure)	Imaging, 3D modeling	Cobb angle; axial rotation; vertebra identified (such as T1-L5); coronal balance	Cross validation not otherwise specified	49	Sensitivity = 100% Specificity = 75% Pearson *r* coefficient = 0.3–0.9 PPV = 80–81% NPV = 82–100%	Neural-network analysis of full-torso scan imaging shows promise to accurately estimate scoliotic spinal deformity in a variety of patients, without radiation exposure
Samadi [50] 2022	SVM, KNN decision tree/random forest	Radiographic analysis (classification of curve type and severity, decreased radiation exposure)	Imaging	Cobb angle; coronal balance	FCV	30	Accuracy = 91.4% Precision (PPV) = 89.5% Recall (Sensitivity) = 91.4% F1 Score = 89.3%	The authors developed an efficient radiation-free machine learning method using intervertebral efforts during gait to classify the severity of AIS
Jiang [51] 2022	CNN	Radiographic analysis (classification of curve type and severity, decreased radiation exposure)	Imaging and 3D modeling	Not specified	FCV	102	F1 Score = 79.5% Sensitivity = 78.5% PPV = 79.5% Jaccard = 66.8%	The authors propose a novel spinal ultrasound image segmentation network called UGBNet, which can accurately segment and identify bony features, such as spinous and transverse processes, in spinal ultrasound images to guide AIS treatment
Yang [52] 2019	CNN	Radiographic analysis (curve severity, decreased radiation exposure), detection/diagnosis	Imaging, live camera	Cobb angle: vertebra identified (such as T1-L5)	FCV	3240	AUC = 0.95 Sensitivity = 87.5–95.1% Specificity = 83.5–85.7% Accuracy = 75–80%	The authors showed that DLAs can be trained to detect scoliosis, identify cases with a curve ≥ 20°, and perform severity grading using unclothed back images with an accuracy, sensitivity, specificity, and PPV that are higher or comparable to those of human experts
Shen [53] 2020	CFCM	Radiographic analysis (curve classification/severity)	3D modeling	Cobb angle; Kyphosis angle; Lumbar Lordosis angle; axial rotation; apical vertebra	Cross validation not otherwise specified	952	N/A	The authors presented a new method of classifying AIS based on a fuzzy clustering algorithm using parameters describing the 3D characteristics of the spinal deformity
García-Cano [54] 2018	Decision tree/random forest	Radiographic analysis (curve classification/severity)	Imaging, 3D modeling	Axial rotation; vertebra identified (such as T1-L5)	FCV	962	Accuracy = 76–78%	The authors’ main contribution in this work is to present the leave-one out and the fan leave-one-out angle measurement techniques, which automatically determine the position of each vertebra with respect to its neighbors, allowing for identification of curve type
Phan [55] 2013	ANN and SOM	Radiographic analysis (curve classification/severity), clinical decision making	Imaging	Cobb angle	Cross validation not otherwise specified	1776	Topological error = 0.02	Self-organizing map classification of AIS could be valuable to surgeons because it bypasses the limitations imposed by rigid classification such as cutoff values on Cobb angle to define curve types
Adankon [56] 2012	SVM	Radiographic analysis (curve classification/severity, decreased radiation exposure)	Imaging, live camera, 3D modeling	Cobb angle	LOOCV	165	Accuracy = 95%	The authors proposed to build a computer-aided diagnosis system which can classify the scoliosis curve type using 3D back surface images which are obtained from noninvasive acquisitions, decreasing radiation exposure
Mezghani [57] 2012	ANN and SOM	Radiographic analysis (curve classification/severity), clinical decision making	Imaging	Cobb angle	Not specified	1776	Accuracy = 88%	The authors trained a network using a database of Cobb angle measurements which resulted in two spatially matched maps, one of Lenke classification and the other of fusion region category. The association of the two maps showed that the Lenke class coincides with the proper fusion level category except at the borders between classes. Surgery planning could benefit from such map associations
Wong [58] 2022	CNN	Imaging analysis (spinal segmentation, automatic measurement)	Imaging, 3D modeling	Cobb angle	Not specified	70	MAD = 3.2–4.2 degrees SEM = 0.8–1.9 degrees ICC = 66–91%	A novel 3D CNN-based algorithm for automatically detecting laminae on spinal ultrasonographs of children with AIS was developed for Cobb angle measurement
Antico [59] 2021	CNN	Imaging analysis (spinal segmentation, decreased radiation exposure), detection/diagnosis	Imaging	Cobb angle; axial rotation; horizontal translation; apical vertebra; vertebra identified (such as T1-L5)	FCV	25	F1 Score = 87%	The authors proposed a data-efficient method to automatically segment the thoracic spine (T5-T12) using a CNN trained with MRI images. The proposed algorithm can be considered as the first step towards MRI-based screening for AIS
Wu [60] 2018	CNN	Prediction (Cobb angles), radiographic analysis (automatic measurement)	Imaging	Cobb angle; Kyphosis angle; Lumbar Lordosis angle; vertebra identified (such as T1-L5)	FCV	526	CMAE = 4.0 degrees for Cobb Angle	When validated on the authors’ large dataset of 526 x-ray images, the MVC-Net was able to achieve automated estimation of Cobb angles in both AP and LAT x-rays
Ishikawa [61] 2023	CNN	Prediction (Cobb angles)	Imaging, live camera; 3D modeling/scanning/surface topography; patient characteristics	Cobb angle	FCV	100	CC = 87% MAE = 4.7 degrees RMSE = 6.0 degrees	The performance of the DLA with a 3D depth sensor was validated for predicting the Cobb angle using an independent external validation dataset. Performance did not significantly vary based on if patient was clothed or unclothed
Alfraihat [5] 2022	ANN, SVM, decision tree/random forest	Prediction (Cobb angles and AIS progression)	Imaging, patient	Cobb angle; Kyphosis angle; Lumbar Lordosis angle; axial rotation; Wedge angle; vertebra identified (such as T1-L5)	FCV	145	MAE = 4.6 degrees	The authors demonstrated they can predict the final major Cobb angle value within 5 degrees error from 2D radiographic features. Such methods could be directly applied to guide intervention timing and optimization for AIS treatment
Kokabu [26] 2021	CNN	Prediction (Cobb angles), detection/diagnosis	Imaging, patient characteristics	Cobb angle	FCV	160	Accuracy = 84–94% MAE = 4 degrees RMSE = 5.6 degrees	The three-dimensional depth sensor imaging system with its newly innovated CNN for regression is objective and has significant ability to predict the Cobb angle in children and adolescents with AIS
Watanabe [62] 2019	CNN	Prediction (Cobb angle), radiographic analysis (automatic measurement), clinical decision making	Imaging, 3D modeling	Cobb angle	Not specified	1,996	MAE Cobb = 3.6 pixels MAE AVR = 2.9 degrees	The authors’ proposed method of estimating the Cobb angle and AVR from moiré images using a CNN is expected to enhance the accuracy of scoliosis screening
Zhang [63] 2017	CNN	Prediction (Cobb angles)	Imaging, 3D modeling	Cobb angle	Cross validation not otherwise specified	235	ICC = 81–98% MAD = 2.9–5.4 degrees	Vertebral slopes can be predicted by DNN. The computer-aided system can be used to perform automatic measurements of Cobb angle, which is used to make reliable and objective assessments of scoliosis
Yahara [24] 2022	CNN	Prediction (AIS progression), clinical decision making	Imaging	Cobb angle; Kyphosis angle; Lumbar Lordosis angle	LOOCV	58	Accuracy = 69% AUC = 0.6 Sensitivity = 61% Specificity = 77%	The deep learning system developed could predict the progression of scoliosis with the highest accuracy of 69% and an AUC of 0.7. In addition, the CNN could predict the progression of scoliosis with higher accuracy than that of spine surgeons
Wang [64] 2021	CNN	Prediction (AIS progression), clinical decision making	Imaging	Cobb angle; Kyphosis angle; Lumbar Lordosis angle; apical vertebra; coronal balance	FCV	438	Accuracy = 74.9–78.1% Sensitivity = 71.9–76.3% Specificity = 78.8–81.9%	This is the first report of automated prediction of AIS curve progression based on radiomics and deep learning. Patients predicted to be at-risk of progression may be counseled to receive early bracing
Tajdari [65] 2021	CNN	Prediction (AIS progression and clinical outcomes)	Imaging, patient characteristics	Cobb angle; vertebra identified (such as T1-L5)	Cross Validation not otherwise specified	190	Not specified	The proposed work combines biomechanics, finite element analysis, advanced data science and image analysis technique to develop a predictive, patient-specific model for spine curvature in AIS patients
Ghaneei [66] 2019	KNN	Prediction (AIS progression), radiographic analysis (curve severity, decreased radiation exposure)	Imaging, 3D modeling	Cobb angle	Not Specified	128	Curve severity accuracy = 72–80% Curve severity sensitivity = 81–93% Curve severity specificity = 53–79% Curve progression accuracy = 93% Curve progression sensitivity = 83% Curve progression specificity = 95%	The authors utilized a KNN algorithm to predict AIS curve severity and progression based on surface topography monitoring and have shown that this method has the potential to significantly reduce the number of X-rays required during clinical follow-up of patients with AIS
García-Cano [67] 2018	Decision tree/random forest	Prediction (AIS progression, Cobb angle)	Imaging, 3D modeling	Cobb angle; vertebra identified (such as T1-L5)	FCV	150	RMSE Cobb = 1.83–5.18 degrees	The results obtained from the authors’ approach indicate that predictions based on independent components are very promising. Independent component analysis offers the means to identify the variation in the 3D space of the evolution of spinal curvature
Kadoury [68] 2017	KNN	Prediction (AIS progression, Cobb angle)	Imaging, 3D modeling	Cobb angle; Kyphosis angle; Lumbar Lordosis angle; axial rotation	LOOCV, FCV	745	Accuracy = 81% Sensitivity = 87.9% Specificity = 75.3% AUC = 0.85	The authors achieved a higher prediction accuracy and improved the modeling of spatiotemporal morphological changes in highly deformed spines compared to other learning methods. The proposed framework can process the entire spine model which adds significant insight on the predominant features used for the prediction of curve progression
Chalmers [69] 2015	CFCM	Prediction (AIS progression) clinical decision making	Imaging, patient characteristics	Cobb angle; apical vertebra	FCV	62	Sensitivity = 100% Specificity = 69% PPV = 71% NPV = 100% MCC = 70%	In predicting brace treatment outcome, the authors’ computer model outperformed most experts individually and was comparable to experts as a group. This provides some justification for using this type of model in a decision support system
Ajemba [70] 2005	SVM	Prediction (AIS progression)	Imaging, patient characteristics	Cobb angle; axial rotation; apical vertebra; vertebra identified (such as T1-L5); T1-tilt	FCV	44	Accuracy = 65–80% Sensitivity = 86% Specificity = 67% PPV = 67% NPV = 86%	The authors demonstrated that it may be possible to predict the risk of progression of AIS (to an accuracy of up to 80%) from radiographic indicators and clinical variables using a decision support system based on a Support vectors classifier
Wu [71] 2022	CNN	Detection/diagnosis, radiographic analysis (automatic measurement)	Imaging	Cobb angle; apical vertebra	Not specified	788	Accuracy = 77.2%-89.4% Sensitivity = 84.1%-97.4% MAE = 1.0–1.8 degrees	A DLA model for X-ray images was successfully established and achieved high sensitivity and accuracy for scoliosis recognition. MSE-Net could effectively assist radiologists in the diagnosis of AIS
Vergari [72] 2020	CNN	Detection/diagnosis	Imaging	Not specified	FCV	796	Accuracy = 96.9–98.3%	The proposed classification model, the originality of which is the coupling of a CNN with discriminant analysis, can be used to automatically label radiographs for the presence of scoliosis treatment
Jamaludin [73] 2020	CNN	Detection/diagnosis, imaging analysis (decreased radiation exposure)	Imaging, patient characteristics	Not specified	Not specified	449	Sensitivity = 86.5% Specificity = 96.9% AUC = 0.80 Kappa = 90%	The authors developed an accurate and valid automated method for identifying and quantifying spinal curvature from total body DXA scans
Meng [27] 2022	CNN	Clinical decision making, prediction (Cobb Angles), radiographic analysis (curve type severity)	Imaging	Kyphosis angle; Lumbar Lordosis angle; coronal balance	Not specified	1079	Sensitivity = 95.7%-97.4% Specificity = 88.1%-98.4% PPV = 95.4%-96% NPV = 87.1%-98.8% Accuracy = 93.8%-97.9%	The authors deployed the first prospectively validated auto-alignment analysis model for spine curve classification using an open platform. The AI-powered hybrid system, SpineHRNet + was trained and assessed with radiographs with variable qualities and sources
Pasha [74] 2021	Decision tree/random forest	Clinical decision making/treatment outcome prediction	Imaging, 3D modeling, patient characteristics	Kyphosis angle; Lumbar Lordosis angle; apical vertebra; vertebra identified (such as T1-L5); T1-tilt	Not specified	371	Accuracy = 75% AUC = 0.79–0.86	Preoperative patient-specific parameters in a cohort of AIS (64 variables) who received PSF predicted the two-year spinal alignment clusters outcomes at 64% accuracy. Adding the surgical factors improved the outcome prediction to 75%
Mandel [75] 2021	CNN	Clinical decision making/treatment outcome prediction	3D modeling	Cobb angle	LOOCV	72	Accuracy = 86.8% RMSE = 1.7 degrees F1 Score = 95.1%	The authors presented a spine surgery outcome prediction method for AVBT procedures, learning the physiological variations between sequential biplanar examinations which allows for the production of 3D spine models at 1 and 2 years following the tethering application
Peng [76] 2020	Decision tree/random forest	Clinical decision making/ treatment outcome prediction	Imaging, patient characteristics	Cobb angle; Kyphosis angle; Lumbar Lordosis angle; vertebra identified (such as T1-L5); T1-tilt	LOOCV	44	Accuracy = 90.9% AUC = 0.94 F1 Score = 66.7%	The random forest using synthetic minority oversampling techniques has value for predicting the individual risk of developing proximal junctional kyphosis after long instrumentation and fusion surgery in Lenke 5 AIS patients

*AI* artificial intelligence, *AIS* adolescent idiopathic scoliosis, *ANN* artificial neural network, *AUC* area under the curve, *AVBT* anterior vertebral body tethering, *AVR* axial vertebral rotation, *CC *correlation coefficient, *CFCM* conditional fuzzy c-means, *CMAE* circular mean absolute error, *CNN* convolutional neural network, *CT* computed tomography, *DNN* deep neural network, *DLA* deep learning algorithm, *DXA* dual-energy x-ray absorptiometry, *ICC* inter-class coefficient, *FCV* fold-cross validation; *KNN* k-nearest neighbor, *L* lumbar, *LAT* lateral, *LOOCV* leave-one-out cross validation, *MAD* mean absolute difference, *MAE* mean absolute error, *MCC* Matthew’s correlation coefficient, *MRI* magnetic resonance imaging, *MSE*: mean squared error, *MT* mid-thoracic, *NPV* negative predictive value, *PA* posterior-anterior, *PPV* positive predictive value, *PSF* posterior spinal fusion, *PT* proximal-thoracic, *RMSE* root mean squared error, *SEM* standard error of the mean, *SMAPE* symmetric mean absolute percentage error, *SOM* self-organizing map, *ST* surface topography, *SVM* support vector machine, *TL* thoracolumbar

The average patient sample size used to train the AI was 503 patients with a mean age of 13.8. The mean Cobb angle across all studies was 30.4º, with an average minimum of 11.4º and average maximum of 62.7º. The mean performance accuracy of the AI models across studies which reported it (52.5% of studies) was 85.4% with an average validation set of 282 samples (of studies which reported validation sample size).

### AI parameters

**Fig. 4:**
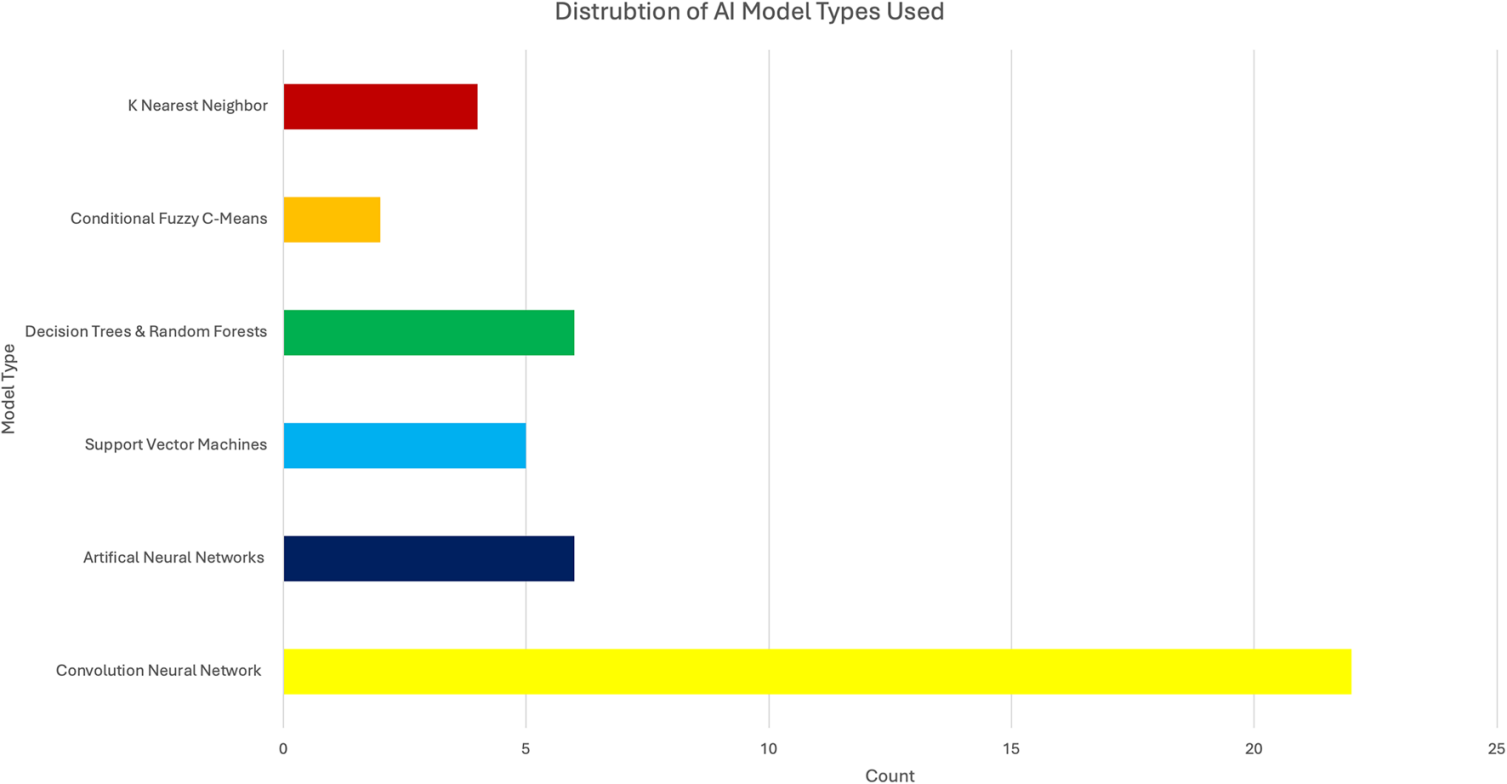
Distribution of AI model types used in included studies

The most frequently used ML techniques were CNNs (22/40; 55%), decision trees and random forests (6/40; 15%), ANNs (6/40; 15%), support vector machines (5/40; 12.5%), K nearest neighbor (4/40; 10%), and conditional fuzzy c-means (2/40; 5%) (Fig. 4). Nearly all studies used radiographic imaging (37/40; 92.5%) and 42.5% of studies also used 3D modeling of the spine as an input variable. Only 9 studies (22.5%) accounted for skeletal maturity by implementing either the Risser or Sanders stage as an input variable in their ML model. The major radiographic features extracted were the Cobb angle (32/40; 80%), the apical vertebral level (10/40; 25%), the lumbar lordosis angle (10/40; 25%), and axial vertebral rotation (8/40; 20%). The major validation techniques were k-fold cross-validation (16/40; 40%), other unspecified forms of cross-validation (8/40; 20%),leave-one-out cross-validation (5/40; 12.5%), and 12 studies (30%) did not report any validation method.

Model performance metrics included accuracy/percent correct (21/40, 52.5%), sensitivity (15/40, 37.5%), specificity (11/40, 27.5%), mean absolute error (MAE) (78/40, 17.5%), positive predictive value (PPV) (7/40, 17.5%), area under the curve (AUC) (6/40, 15%), F1 score (6/40, 15%), root mean squared error (RMSE) (5/40, 12.5%), negative predictive value (NPV) (4/40, 10%), inter-class correlation coefficient (4/40, 10%), mean absolute difference (2/40, 5%), kappa statistic (1/40, 2.5%), Matthew’s correlation coefficient (1/40, 2.5%), Pearson correlation coefficient (1/40, 2.5%), and Jaccard index (1/40, 2.5%). The majority of studies (65%) evaluated their AI model with multiple performance metrics.

### AI application in AIS

**Fig. 5:**
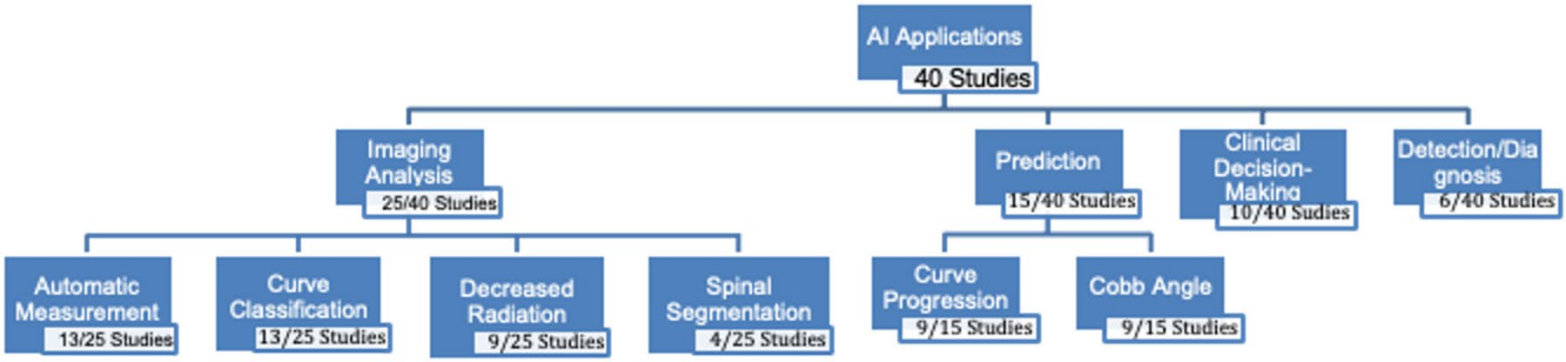
Mapping the landscape: AI applications in AIS

The majority of AI applications in AIS were used for imaging analysis (25/40; 62.5%). Within imaging analysis, automatic measurement (13/25; 52%), curve classification/curve severity (13/25; 52%), decreased radiation exposure (9/25; 36%) and spinal segmentation (4/25; 16%) were the most common sub-applications. Prediction was the second most common application (15/40; 37.5%). Studies including the prediction of AIS were split into two main categories: prediction of AIS progression (9/15; 60%), and prediction of Cobb angles (9/15; 60%). The last major applications of AI in AIS were clinical decision support, such as treatment decisions, which included treatment management (surgery vs. bracing) and procedural selection (10/40; 25%), and detection/diagnosis of AIS (6/40; 15%) (Fig. 5). 25 studies (62.5%) did not report practical strategies or guidelines on clinical implementation of their AI technique for AIS management.

## Discussion

This scoping review identified key AI techniques, input/output features, applications in AIS management, along with gaps in the current literature. The most frequently utilized ML algorithms included CNNs, decision trees and random forests, and ANNs. CNN models were commonly trained on radiographic imaging to automate measurements such as the Cobb angle, lumbar lordosis, and axial vertebral rotation, and to classify curves by type and severity using the Lenke classification. Other common AI applications included predicting Cobb angle through non-radiological images (e.g. ultrasound, gait analysis) and predicting curve progression based on patient-specific factors. Less common applications involved diagnosing AIS and assisting with prognostic estimations and treatment decisions (surgery vs bracing). Despite the promise of these applications, several shortcomings exist in current AI research for AIS. Notably, there was a lack of practical clinical implementation recommendations and external validation in the examined studies. This, combined with the opaque nature of AI, may limit clinician trust and hinder AI integration into everyday clinical workflows. The AI models described should supplement rather than replace the diagnostic and treatment decisions of orthopedic surgeons and radiologists. This review helps orthopedic spinal surgeons understand how AI is currently applied in AIS management, while highlighting the need for future research including clinical implementation of these models.

The primary application of AI in AIS management is the use of CNNs to automatically analyze radiographic and other diagnostic imaging, as evidenced by over 60% of the included studies. In a retrospective cohort study by Berlin et al. [20] a fully automated CNN model measured several radiographic parameters including T1-tilt, clavicle angle (CA), coronal balance (CB), Cobb angles, and lumbar modifier (LM), for 100 AIS patients. These measurements were compared to those from two experienced physicians, considered the ground truth. The model demonstrated interclass correlation coefficient’s ranging from 0.78 to 0.98 (highest for CA and CB) and had root mean squared error (RMSE) values comparable to those of the physicians. Although the study did not mention practical guidelines for clinical implementation, the proposed algorithm analyzed an image in 15 s, compared to 3–7 min for the physicians. This highlights the algorithm’s potential to supplement physicians in routine, time-consuming measurements and could be applied to large-scale datasets for research purposes.

In other studies applying AI for automatic imaging analysis, Jiang et al*.* [21] used ultrasound images and their UGBNet model to automatically conduct spinal segmentation and identify key anatomical landmarks such as spinal and transverse processes. Their two-step model first identifies and segments bony landmarks on ultrasound and then creates a 3D reconstruction of the patient’s spine, allowing for curve visualization without radiation exposure. Additionally, Samadi et al*.* [22] used lumbosacral joint effort during gait from 30 patients, and an ensemble voting classifier to categorize mild scoliosis (10 < Cobb angle < 25), moderate scoliosis (25 < Cobb angle < 45) and severe scoliosis (Cobb angle > 45), with 91% accuracy. Despite the lack of explicit guidelines for clinical implementation and small sample sizes, these studies offer promising preliminary analysis that could eliminate the need for radiation exposure from serial EOS films in the future. Although EOS imaging moderately decreases radiation exposure compared to traditional radiographs, eliminating radiation exposure entirely with accurate imaging analysis from ultrasound or gait would be an even more significant improvement [23].

Prediction of curve progression and Cobb angle is a significant application of AI in AIS management, with nearly 40% of studies performing this task. Yahara et al. [24] used a deep CNN and radiographs to categorize AIS patients with an initial Cobb angle of 10–25 degrees into progression (increase of 10 or more degrees at 2-year follow-up) and non-progression groups (less than a 5-degree increase). The model achieved 69% accuracy and an AUC of 0.70, outperforming five physicians it was compared to, but falling short of clinical implementation standards. This model only used radiographic data and excluded clinical parameters such as age, sex, growth rate, and skeletal maturity, and it was trained on just 58 subjects. In another study, Alfraihat et al. [5] compared three models—ANN, random forest, and support vector machine—to predict the final Cobb angle in 193 AIS patients. This model integrated radiographs with clinical parameters such as sex, Lenke curve type, brace status, and Risser + stage, achieving a MAE of 4.6 degrees for the final Cobb angle. Notably, this was one of the nine studies to include skeletal maturity in their model, something we encourage future models to do as well. The authors created a publicly accessible web app and reported input feature weights, enhancing transparency and usability. However, they could not prospectively validate this model across multiple institutions, limiting its generalizability. Models that can accurately predict curve progression and Cobb angle can supplement a scoliosis specialist’s clinical judgment, aiding decisions on conservative versus aggressive treatment in borderline AIS patients. Although bracing can reduce curve progression, it often negatively impacts quality of life by restricting activities, causing conflicts at school, and decreasing socialization [25]. These models could help clinicians avoid unnecessary bracing and its associated impairments by identifying patients unlikely to progress.

Surprisingly, only 15% of studies applied AI for detecting and diagnosing AIS. Kokabu et al. [26] retrospectively used a CNN with data from 3D depth sensors on the backs of 160 AIS patients to predict Cobb angles and diagnose AIS if the angle was greater than 10 degrees. The model achieved a MAE of 4.4–4.7 degrees and an RMSE of 5.8 to 6.3 degrees. At a Cobb angle of 10 degrees, it reached 94% accuracy, 99% sensitivity, and a PPV of 95%, showing clinically acceptable diagnostic power without radiographs. A notable strength of this study is its validation across five institutions, enhancing model generalizability. Given the scarcity of literature utilizing AI for AIS diagnosis, future studies should adopt a similar approach, emphasizing prospective, multi-institutional clinical validation.

Most (62.5%) of the examined studies did not address the clinical implementation of their AI models, highlighting a significant gap in current research. Meng et al. [27] provide an exemplary model by detailing the clinical implementation of their SpineHRNet + model, which detected spinal curvature (95.7% sensitivity, 88.1% specificity) and classified curve severity (88.6–90.8% sensitivity). They prospectively validated their model on 337 AIS patients and outlined the clinical workflow of their AI platform, AlignProCare. Their workflow involves uploading spinal radiographs via smartphone or computer to a data center for processing and anonymizing. The AI-powered SpineHRNet + model segments the images and sends them back to the clinician for review. The authors also provided an online platform explaining the process to potential patients. To address the deficiency in clinical implementation guidelines, future AI in AIS studies should explicitly delineate clinical workflows, as done by Meng et al. This can include illustrations in manuscripts, using smartphone technology and apps, creating public websites, and ensuring AI model compatibility with existing electronic health record (EHR) systems. There are several reporting guidelines, including the Consolidated Standards of Reporting Trials-Artificial Intelligence (CONSORT-AI) [28], we endorse future studies to utilize. AI holds promise for revolutionizing healthcare efficiency and reducing future costs, but the current implementation cost, which can exceed 1 million dollars for a single model, including development, infrastructure, and staff education, is often overlooked [29]. Developing tailored AI algorithms requires time and funding for data acquisition, model training, and integration into EHRs. Other costs include staff training, IT infrastructure, data security, and model updates [30]. Additionally, regulatory and ethical barriers include informed consent, human oversight, job displacement, and commercial interests versus equitability [31]. Regulatory barriers, such as FDA approval [31], are challenging since AI is designed to continually update, unlike the static devices typically assessed by the FDA. Overall, practical, financial, ethical, and regulatory barriers must still be navigated.

A potential drawback of large-scale AI algorithms is their “black box” nature. Many ML technologies continuously refine their internal decision-making structures in ways even their programmers cannot fully grasp, much less healthcare practitioners [32]. While practitioners can compare and validate AI model outcomes, they often do not understand how the algorithm arrives at its final diagnosis. This black box nature can introduce bias, accountability issues, and decreased trust from clinicians and patients [8, 31, 32]. To address this, efforts are being made to develop explainable artificial intelligence (XAI), which can illustrate how input variables influence AI predictions. For example, Shapley Additive explanations (SHAP) use game theory by applying principles of efficiency, symmetry, dummy, and additivity to show how each input variable contributes to the model’s final decision [33]. SHAP has been used in various clinical scenarios, such as identifying key lab values for predicting GI bleed mortality [34]. Gradient-weighted class activation mapping (GradCAM) is another XAI tool, primarily used with CNNs for medical imaging predictions [33]. GradCAM can formulate heat maps that help the provider understand which parts of the image(s) contributed most to the CNNs prediction, which is useful in applications like evaluating chest radiographs for COVID-19 diagnosis [35] or assessing CT images [36] for acute intracranial hemorrhage detection. XAI holds promise for increasing AI model transparency, thus facilitating clinician understanding and trust. Therefore, we recommend future studies on AI in AIS management to incorporate XAI where feasible.

Lastly, the average performance and accuracy of the evaluated models were not adequate for safe clinical implementation. To improve model performance, it is crucial to increase the amount and diversity of training data through large-scale, multi-institutional studies. Balagurunathan et al. [37] provide a useflow flow chart highlighting AI model development, from early cohort studies with limited sample sizes and training data to large, prospective studies, and eventually to national, large-scale adoption studies. Another promising route for increasing AI model performance is through the creation of multimodal models that integrate tabular data (i.e. labs, demographics, medical history, etc.), free-text from clinical notes utilizing natural language processing (NLP), image data (radiographs, pathology, MRI, CT, etc.), and time-series data such as physical exam information from wearable sensors [38]. Multimodal AI models have been implemented in numerous specialities [39–41] and have demonstrated potential for increasing accuracy 1.2–27.7% compared to single-modality systems. These models mimic the clinician’s thought process by evaluating diverse data types, holding promise for enhancing AI model performance in the future. AI will never be perfect and is prone to missing unconventional cases, such as unusually rapid progression of AIS during growth or late progression of AIS after cessation of growth. Therefore, one must remember AI serves as an additional tool in a clinician’s repertoire, rather than replacing the clinician altogether.

This scoping review has several limitations. The variability in study design, interventions, and populations among the 40 included studies made a pooled analysis impractical. As a result, we examined results independently, since a comparison of results, as would be done in a systematic review or meta-analysis, was not feasible. However, because our objective was to map the applications of AI in AIS, a pooled analysis was not necessary. While this review aimed to provide a comprehensive overview, it is possible that relevant studies were missed due to publication bias or limitations in our search strategy. Furthermore, the rapid pace of AI development means that new advancements may have emerged since the time of this review, potentially affecting the relevance of the findings.

## Conclusion

This scoping review found that AI in AIS management is primarily used for automatic imaging analysis, curve classification, and prediction of curve progression. Less common applications included treatment decisions and AIS diagnosis. Notably, many studies lacked practical clinical implementation recommendations, external validation, and model transparency. These issues, combined with the “black box” nature of AI, and regulatory, financial, and ethical barriers, may hinder AI integration into clinical workflows. We recommend future studies integrate XAI, report clinical implementation guidelines, and adhere to CONSORT-AI or similar standardized reporting guidelines. While model performance varied greatly, future models may benefit from the integration of multiple input data modalities (tabular data, clinical notes, imaging, physical exam). Ultimately, this scoping review serves as a first step to outline the current landscape of AI in AIS literature, necessitating future systematic reviews and meta-analyses to provide more specific recommendations for addressing deficiencies in this field.

## Supplementary Information

Below is the link to the electronic supplementary material.

Supplementary file1 (DOCX 14 KB)

## Data Availability

All data is available upon reasonable request to the corresponding author.
