# Supplementary material for: Applications of artificial intelligence for adolescent idiopathic scoliosis: mapping the evidence
Source: Spine Deform. 2024 Aug 17;12(6):1545–70. doi: 10.1007/s43390-024-00940-w (PMC11499369; doi:10.1007/s43390-024-00940-w)
Supplement: Supplementary file 1 — Supplementary file1 (DOCX 14 KB) [file 43390_2024_940_MOESM1_ESM.docx]

(“AIS” [tiab] OR “Scoliosis” [tiab] OR “Adolescent Idiopathic Scoliosis”[tiab] AND (“Artificial Intelligence”[Mesh] OR “Machine Learning”[Mesh] OR “Supervised Machine Learning”[Mesh] OR “Deep Learning”[Mesh] OR “support vector machine”[MeSH Terms] OR “support vector machine”[All Fields] OR “Support Vector Machine”[Mesh] OR naive bayes[tiab] OR “bayesian learning”[tiab] OR neural network*[tiab] OR “support vector”[tiab] OR support vectors[tiab] OR random forest[tiab] OR “deep learning”[tiab] OR “machine prediction”[tiab] OR “machine intelligence”[tiab] OR “computational intelligence”[tiab] OR “computational learning”[tiab] OR “computer reasoning”[tiab] OR “machine learning”[tiab] OR convolutional network*[tiab] OR “artificial intelligence”[tiab])

"Adolescent Idiopathic Scoliosis"[Tiab] AND ("Artificial Intelligence"[Mesh] OR "Machine Learning"[Mesh] OR "Supervised Machine Learning"[Mesh] OR "Deep Learning"[Mesh] OR "support vector machine"[MeSH Terms] OR "support vector machine"[All Fields] OR "Support Vector Machine"[Mesh] OR naive bayes[tiab] OR "bayesian learning"[tiab] OR neural network*[tiab] OR "support vector"[tiab] OR support vectors[tiab] OR random forest[tiab] OR "deep learning"[tiab] OR "machine prediction"[tiab] OR "machine intelligence"[tiab] OR "computational intelligence"[tiab] OR "computational learning"[tiab] OR "computer reasoning"[tiab] OR "machine learning"[tiab] OR convolutional network*[tiab] OR "artificial intelligence"[tiab])
